# Inappropriate Fiddling with Statistical Analyses to Obtain a Desirable P-value: Tests to Detect its Presence in Published Literature

**DOI:** 10.1371/journal.pone.0046363

**Published:** 2012-10-08

**Authors:** Gary L. Gadbury, David B. Allison

**Affiliations:** 1 Department of Statistics, Kansas State University, Manhattan, Kansas, United States of America; 2 Department of Biostatistics, University of Alabama at Birmingham, Birmingham, Alabama, United States of America; Cardiff University, United Kingdom

## Abstract

Much has been written regarding p-values below certain thresholds (most notably 0.05) denoting statistical significance and the tendency of such p-values to be more readily publishable in peer-reviewed journals. Intuition suggests that there may be a tendency to manipulate statistical analyses to push a “near significant p-value” to a level that is considered significant. This article presents a method for detecting the presence of such manipulation (herein called “fiddling”) in a distribution of p-values from independent studies. Simulations are used to illustrate the properties of the method. The results suggest that the method has low type I error and that power approaches acceptable levels as the number of p-values being studied approaches 1000.

## Introduction

*If one in twenty does not seem high enough odds, we may, if we prefer it, draw the line at one in fifty (the 2 per cent. point), or one in a hundred (the 1 per cent. point). Personally, the writer prefers to set a low standard of significance at the 5 per cent point, and ignore entirely all results which fail to reach this level. A scientific fact should be regarded as experimentally established only if a properly designed experiment rarely fails to give this level of significance.*

*–* Sir Ronald Aylmer Fisher (1926), The Journal of the Ministry of Agriculture.

The interpretation of “statistical significance” as a p-value equal to or less than 0.05 has been attributed to the above well-known quotation from R.A. Fisher and other statements by him. In 1982, however, Cowles and Davis [1] presented a thorough treatment of the origins of the 0.05 level of statistical significance and suggested that this threshold may in fact have predated Fisher. Regardless of its origin, one cannot help but notice, from journal articles and in working with scientists from other fields, that the 0.05 threshold for significance holds to this day. Results with p-values below 0.05 are hailed as “significant findings.”

The tendency for researchers to favor submission of significant findings for publication over insignificant ones, and the tendency for journals to favor publishing these as well, is behind the well-known issue of publication bias in meta-analysis (see Rothstein et al. [2] for an extensive discussion). Emerson et al. [3] conducted a study showing that peer-reviewed manuscripts reporting statistically significant findings were rated as methodologically stronger than were those with findings that were not significant, even when the methods used in both papers were identical.

In 2007 Ridley et al. [4] investigated journal reporting bias, that is, the tendency for smaller p-values rather than larger ones to be reported in journals. Several citations therein referred to selective reporting bias and the tendency for statistically significant findings to have a higher chance of publication and to be published more quickly (e.g., see Stern and Simes [5]). In Ridley et al. [4], four subintervals within the usual statistical significance range of 0–0.05 were considered: [0.01, 0.05), [.001, 0.01), [0.0001, 0.001), and below 0.0001. The values separating the intervals are common significance thresholds for which p-values are often rounded and reported as simply being below the threshold, rather than being numerically reported [4]. The rationale to the Ridley et al. [4] study was that, in the presence of selective publication, a disproportionately larger number of p-values would be reported just below the interval threshold rather than above. Over 3000 reported p-values were collected for their study–over 1000 from each of three prominent science journals (2003 and 2004 editions): *Proceedings of the Royal Society of London, Series B*; *Nature*; and *Science*. A key challenge in their technique was to model a null situation in which no reporting bias was present. To address this, they divided each subinterval into two halves and, assuming that the distribution of p-values should be smooth, used three different statistical models to compute an expected proportion of p-values in each half of each subinterval if no selective reporting had occurred. By comparing the observed proportion to the expected proportion in these subintervals, they conducted a test of whether selective reporting had occurred. Their findings suggested, regardless of which of the three methods was used to report the expected proportions of p-values and across all three journals, that an unexpectedly high proportion of p-values was reported just below the threshold for each interval. These findings, they concluded [4], were more likely due to biased reporting than to any artifact resulting from their proposed method of analysis.

Herein we consider an issue somewhat analogous to the one considered by Ridley et al. [4] but distinctly different in its focus. We consider the situation in which an investigator obtains a p-value of just above 0.05, say, 0.06. Intuition would suggest that a tendency may exist to conduct one or more alternative analyses involving transformations, removal of outliers, additional terms in a model, etc., in order to “tweak” this p-value to a level (e.g., ≤0.05) that may facilitate getting the work published. Such additional testing contingent on initially observing a p-value just above 0.05 we call “fiddling.” If such fiddling does occur, how would it manifest itself in the literature and how can it be statistically detected? We consider these questions here and investigate the feasibility of different methods for detecting whether “fiddling” has occurred with the use of p-values presented in published, peer-reviewed articles. These methods are not intended to investigate publication bias as is sometimes done in a meta-analysis. We believe that publication bias that is present in a meta-analysis is a separate concept from that of fiddling with results in a specific investigation. The tendency for a journal to publish significant findings (versus non-significant ones) may provide incentive for fiddling. Conversely, fiddling with a result could possibly lead to a paper being published as a result of publication bias that might not have been published otherwise. More discussion comparing fiddling with publication bias is given in a final discussion section.

**Table 1:** Pseudo-Code to Generate p-Values Under the Level 2 Null Hypothesis of No Fiddling^a^.

Pseudo-Code	Comments
Compute 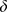 = 1/2	
Compute μ = .8	
Compute σ = .4	
For i = 1 to *N*	*N* is the number of level 1 hypotheses being tested.
Compute B = Bernoulli( 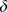 )	
Compute Z = Normal(0,1)	
Compute λ = Normal(μ,σ)	
Compute T = B*Z+(1−B)*(Z+λ)	
Compute p_i_ = 2*(1-CDF_Normal(0,1,|T|))	This is for two-tailed testing.
Next i	

a The code above was implemented in R (www.r-project.org).

Our proposed method involves examining the distributions of p-values reported in collections of literature to detect the inappropriate manipulation of statistical analyses to produce p-values that appear to be significant when the initial analyses produced results that were nearly, but not quite, statistically significant. The focus of the proposed tests is to determine whether there is a noticeable pattern in the number of p-values between 0.05 and 0.075 versus those between 0.075 and 0.1 (any intervals of equal length could be used). P-values are assumed to have been collected from *N* independent studies. As a random variable, a valid p-value has a uniform distribution on the interval 0 to 1 under the null hypothesis, and it has a distribution that should be monotonically decreasing on this interval if the alternative hypothesis is true (see [6] for more discussion of this). Thus, if no fiddling has occurred, there is an “expected relationship” between the number of p-values between 0.05 and 0.075 and those between 0.075 and 0.1. If this relationship is not what is expected, then there is evidence that fiddling has occurred among the studies collected for analysis.

In our method, simulations are used to generate distributions of p-values in which fiddling both did and did not occur. First, p-values are simulated under a null hypothesis of no fiddling. Then, p-values are simulated under an alternative hypothesis that fiddling has occurred. The simulation procedure for generating p-values is described in the next section 2. We then conduct an assessment of the quality of the procedure for simulating p-values. This involves evaluating the sampling variability in the null distribution of p-values and determining whether the alternative distribution (i.e., a distribution for which fiddling has occurred) is detectable as different from the null case. In a fourth section we propose a simple test that can be used by an investigator studying a body of literature that takes a single vector of p-values and evaluates whether there is evidence that fiddling has occurred among the original studies contributing p-values to that vector. Next, we describe a test using a mixture model approach that uses known theoretical properties of p-values as random variables. A final section discusses the proposed method within the broader context of publication bias, the role of fiddling on effect sizes, the number of studies required to carry out the proposed method, and the issues to consider when designing a study for the purpose of investigating fiddling.

### Simulation Procedure

#### 2.1. Simulation under the null hypothesis of no fiddling

Two terms that are used throughout are *level 1 test* and *level 2 test*. By level 1 tests, we refer to the tests of individual null hypotheses in the individual studies, the p-values from which then become the data for the level 2 tests of fiddling. To simulate p-values under the level 2 null hypothesis of no fiddling, we first specify that the level 1 test statistics have a normal distribution under the level 1 nulls, and then we specify a distribution of effect sizes for the level 1 test statistics, given that some of the nulls will be false. Specifically, we set the distribution of the level 1 test statistics to be:


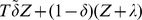
, where 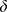 is a mixing parameter between 0 and 1, *Z* represents the standard normal distribution, and λ is a non-centrality parameter that itself can be a randomly distributed variable. For our initial simulations, we set 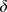 = ½ and let λ∼N(0.8;0.4). The pseudo-code to generate the p-values under this scenario is as shown in Table 1.

**Table 2:** Pseudo-Code to Generate p-Values When the Level 2 Null Hypothesis of No Fiddling is False.

Pseudo-Code	Comments
Compute 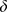 = 1/2	
Compute μ = .8	
Compute σ = .4	
Compute ρ = .95	
Compute α = .05	
Compute ι = .025	
Compute *m* = 4	
For i = 1 to *N*	*N* is the number of level 1 hypotheses being tested.
Compute B = Bernoulli( 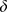 )	
Compute Z = Normal(0,1)	
Compute λ = Normal(μ,σ)	
For r = 1 to *m*+1	
Compute Z_r_ = (ρ^(1/2)^)*Z+ (1-ρ)^(1/2)^*Normal(0,1)	This formula presupposes that ρ is positive.
Compute T_r_ = B*Z_r_+(1−B)*(Z_r_+λ)	
Next r	
Compute Tmax = max(|T_1_|…|T*_m_*_+1_|)	
Compute p_i_ = 2*(1-CDF_Normal(0,1,|T_1_|))	This is for two-tailed testing.
Compute p_min_ = 2*(1-CDF_Normal(0,1,|T_max_|))	
If (p_i_>α AND p_i_≤α+ι) p_i_ = p_min_.	
Next i	

#### 2.2. Simulation when the level 2 null hypothesis of no fiddling is false (i.e., there is fiddling)

In addition to those items specified above, we now need to specify the number of additional tests required when one obtains a p-value in the interval (α,α+ι], where ι is some small positive constant (e.g., 0.025) for a level 1 test. Let us denote this number *m*. We use *m* = 4. Next, we need to specify the correlation structure among the *m*+1 test statistics, which we can denote T_1_ to T_m+1_. The correlation structure used here is compound symmetric with correlation ρ where, initially, ρ = 0.95, reasoning that 0.95 may be a bit conservative (i.e., high) and thereby could cause us to underestimate the sensitivity (power) of our procedure. The pseudo-code to generate the p-values under this scenario is as shown in Table 2.

### Evaluating the Simulated Data

This section describes a scenario in which sampling variability is first assessed by simulating two level 2 null distributions for evaluating the performance of a test by its type I error. When the test is conducted on two null distributions, the test for fiddling should have a type I error close to or below the nominal level (0.05 used here). Next, simulated p-values are obtained from a distribution where fiddling has occurred, and this distribution is tested against one of the null distributions to determine the power of the test. The tests used are simple two-way tests of contingency tables as described in the following testing scenario:

1. Two level 2 null data sets were simulated with *N* tests.
2. A two-way contingency table was generated as shown in Table 3.
3. A chi-square test was conducted and a p-value obtained from a test in which the proportion of p-values between 0.05 and 0.075 was equal in both null distributions. Note that only p-values between 0.05 and 0.1 were used. The p-value from this level 2 test is equivalent to a test in which the row and column categories in Table 3 are independent. The chi-square test may be unreliable when cell counts in the table are small; thus, a second p-value was computed by using Fisher’s exact test for the same hypothesis. Two-tailed p-values were used, but one-tailed p-values could be computed.
4. A level 2 alternative hypothesis data set (fiddling) was simulated and a two-way contingency table was generated as shown in Table 4.
5. The same tests as in 3 were computed for Table 4.
6. The above steps were repeated 5000 times.
7. Power and type I errors were reported for each test.
8. The above procedures were repeated for *N = *400, 600, 800, 1000, and 2000.

**Table 3:** Contingency Table Generated From the Simulation of Two Level 2 Null Data Sets With *N* Tests Each.

	# p-values ∈ (0.05,0.075]	# p-values ∈ (0.075,0.1]
Null 1	N11	N12
Null 2	N21	N22

The results of the testing scenario are shown in Table 5. The results show that p-values are being simulated in such a way that a reasonable test for fiddling maintains a type I error below the nominal level but does not gain substantial power until the number of tests exceeds 1000. We note that Ridley et al. [4] used over 3000 numerically reported p-values as discussed in the Introduction (i.e., they ignored p-values that were rounded below a threshold of significance and reported with a “less than” sign).

### A Simulation Study Evaluating a Test for Fiddling

Data were simulated as described above. The testing described in the prior section used two level 2 null distributions for type I error and a level 2 alternative distribution and a null distribution for power evaluation. The tests described here are different in that a single distribution of p-values was used. The test compares the proportion of p-values in the interval 0.05–0.075 with those in the interval 0.075–0.1. This test for fiddling is appropriate for any situation in which a sufficient number of p-values are obtained from *N* studies. Some indication of how large *N* should be is given in the results that follow. Define the following statistics:

*N = *total number of tests
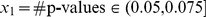


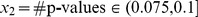


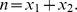
Two different tests are considered below.

### Test 1 was Conducted as Follows

Define the following:
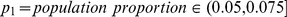


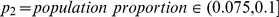



The hypothesis test is 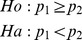 for each simulated distribution of p-values. Note that this test is likely to have a smaller type I error than nominal because it is expected that, in general, the above level 2 null hypothesis will tend to be true in any situation in which some p-values are truly from an alternative distribution of p-values, that is, the level 1 null hypothesis is not true for some level 1 p-values. This is because, as mentioned earlier, the distribution of a level 1 p-value under a null hypothesis is expected to be uniform on the interval 0–1 and is expected to be monotonically decreasing on the interval 0–1 under a level 1 alternative hypothesis. Thus, if a collection of *N* p-values is obtained, some for which the level 1 null hypothesis is true and others for which it is false, it is expected that there will be a larger number of smaller p-values than larger p-values. Estimates of the above parameters are
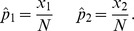



A proportions test using normal approximation with continuity correction was done at a type I error threshold of 0.05 (i.e., the size of the rejection region for this test is 0.05). Note that the two statistics are technically not independent because they both depend on the same *N*. However, given that *N* is substantially larger than 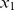 and 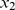, the effect of this dependence should be minimal.

**Table 4:** Contingency Table Generated From the Simulation of a Level 2 Null Hypothesis Data Set and a Level 2 Alternative Hypothesis Data Set (Fiddling).

	# p-values ∈ (0.05,0.075]	# p-values ∈ (0.075,0.1]
Null 1	N11	N12
Alternative	N21	N22

**Table 5:** Results of a Testing Scenario for Evaluating Simulated Data^a^.

*N*	Type I Error (Chi-Sq Test)	Type I Error (Fisher’s Test)	Power (Chi-Sq Test)	Power (Fisher’s Test)
400	0.0250	0.0326	0.1896	0.2308
600	0.0300	0.0384	0.3202	0.3632
800	0.0370	0.0456	0.4494	0.4882
1000	0.0356	0.0418	0.5354	0.5780
2000	0.0366	0.0402	0.8838	0.8952

a Two tests were conducted to evaluate type I error and power for various sample sizes.

### Test 2 was Conducted as Follows

Define the following conditional probability, 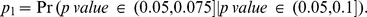



The estimator for this probability is 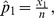 and the level 2 hypothesis test is 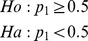. This is a test conditional on the event that a level 1 p-value lies in the interval 0.05–0.1. It is also expected to have a type I error below the nominal error rate because the level 2 null hypothesis is expected to be true in any situation in which some level 1 tests produce p-values from a distribution under a level 1 alternative hypothesis, for the same reason as described above for Test 1. A binomial test was conducted here (i.e., not a normal approximation) using the same size of rejection region as for Test 1.

Type I error and power for the two tests (Test 1 and Test 2) are shown in Table 6. The results from the two tests are nearly the same. The results suggest that it makes little difference whether a test of proportions (out of *N* tests) is done or whether the conditional test that only considers p-values in the interval 0.05–0.1 is used. Both tests are conservative in that type I error rates are below nominal levels. Power begins to rise to acceptable levels at around *N = *800 to 1000 p-values.

**Table 6:** Results of the Performance of Two Tests for Detecting Fiddling in a Distribution of p-Values^a^.

*N*	Type I Error (Test 1)	Type I Error (Test 2)	Power (Test 1)	Power (Test 2)
400	0.0216	0.0216	0.4350	0.4350
600	0.0224	0.0220	0.6132	0.6132
800	0.0208	0.0198	0.7424	0.7416
1000	0.0200	0.0198	0.8222	0.8218
2000	0.0156	0.0152	0.9846	0.9842

a Test 1 considers the total of *N* p-values and Test 2 considers only those in the interval (0.05, 0.1]. Type I error and power for the two tests are reported for various sample sizes.

### A Mixture Model Approach to Testing

#### 5.1. Overview of the procedure

In this approach, p-values resulting from *N* tests are considered and a mixture model of the form below is used to model the p-values,
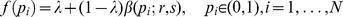
where 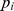 is a p-value for the *i* ^th^ level 1 hypothesis test (out of *N* tests), 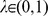 is a weight on the uniform component (i.e., a p-value from a test for which the null hypothesis is true has a uniform distribution), and 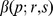 is a beta probability density function (pdf) with parameters *r* and *s*. The beta pdf is used to model p-values from tests for which the level 1 alternative hypothesis is true. This is the model used by Allison et al. [6] to model the distribution of p-values from tests of differential expression in microarray studies. Maximum likelihood estimation is used to fit the model to the *N* p-values.

The primary hypothesis considered here is a level 2 null hypothesis that no fiddling has occurred versus an alternative that fiddling has occurred. If each 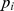 for which the level 1 alternative hypothesis is true is obtained from a valid level 1 statistical test, then the beta distribution will be monotonically decreasing on the interval 0–1. The ability of a beta distribution to serve as a model for the distribution of a p-value under an alternative hypothesis was studied in Hu et al. [7]. An objective function quantifying the fit of a model is the logarithm of the likelihood function computed at maximum likelihood estimates (MLEs) of parameters, given by 
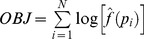
 where 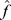 denotes the fitted model. A first claim made here is that, regardless of whether the distribution of p-values contained fiddling or not, the value of *OBJ* will be nearly the same.

**Figure 1:**
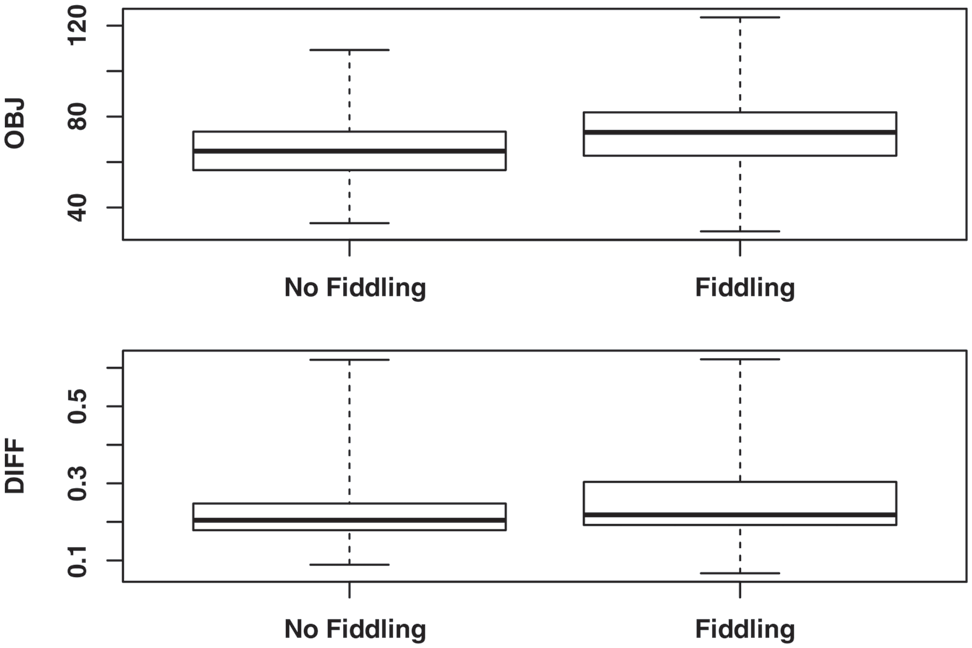
Boxplots of OBJ and DIFF for the conditions of fiddling or no fiddling. Boxplots compare the distributions (from 1000 simulations) of comparison statistics from mixture models fitted to a distribution of p-values for which no fiddling has occurred (i.e., a level 2 null distribution) and to a distribution of p-values for which fiddling did occur. OBJ is the objective function calculated at maximum likelihood estimates of parameters of the model, and DIFF is a difference in the fitted model to 0.05 versus 0.1.

Shown in the top panel of Figure 1 is the boxplot of *OBJ* for 1000 simulated distributions of 1000 p-values each: i.e., where fiddling did not occur and where fiddling did occur. As shown in the figure, the objective function value tends to be slightly higher when fiddling has occurred, but the two distributions have substantial overlap. A second criterion considered was the difference in the value of the fitted model to a p-value equal to 0.05 with that of the fitted model to a p-value equal to 0.1. This difference is denoted as 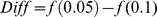 with use of the model notation above. A value of *Diff* was computed for p-values generated under both no fiddling and fiddling. Boxplots for the two distributions are shown in the bottom panel of Figure 1. The values of *Diff* for a distribution of p-values with fiddling tend to be slightly larger than those with no fiddling, but there is substantial overlap of the two sampling distributions of *DIFF*. An assumption that we will make, on the basis of the above simulations, is that a mixture model can be fit to a given collection of p-values, that is, a collection for which it is not known whether fiddling has occurred or not. Furthermore, this fitted model can be used to compute an expected number of p-values in the interval (0.05,0.075] and in the interval (0.075,0.1] when the level 2 null hypothesis of no fiddling is true. This is analogous to the method of Ridley et al. [4], who used the assumed smoothness of a distribution of p-values in a subinterval of 0–0.05 to construct an expected number of p-values in the lower portion and the upper portion of a subinterval under a situation of no reporting bias.

#### 5.2. The proposed testing procedure

Our proposed mixture model approach is as follows:

1. Obtain a collection of p-values from *N* studies.
2. Fit the above mixture model to this distribution of p-values using maximum likelihood estimation. We used the R function “optim.” Numerical optimization procedures do not always converge, and this is somewhat difficult to control in simulation settings, in which thousands of models are fit to simulated data. Mixture models can be particularly challenging. This was studied in detail in Xiang et al. [8]. We found that *N = *400 p-values was large enough to obtain a good fit of the model to simulated distributions of p-values, whether the p-values contained fiddling or not.
3. From the fitted model, obtain the expected number of p-values in the interval (0.05, 0.075] and between (0.075, 0.1]. Call these two numbers E1 and E2, respectively. They are computed by calculating (using an approximation) the cumulative density under the fitted model in the two intervals multiplied by *N*, the total number of p-values.
4. Obtain the two numbers, 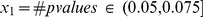 and 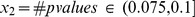.
5. Construct the two-way contingency table as shown in Table 7.
6. Test for fiddling by using a chi-square test or a Fisher’s exact test and report the level 2 P-value from the two tests.

**Table 7:** Two-way Contingency Table for the Mixture Model Approach.

	# *pvalues* ∈(0.05, 0.0075]	# *pvalues* ∈(0.075, 0.1]
Expected	E1	E2
Actual	*x*_1_	*x*_2_

#### 5.3. Properties of the test using simulations

The steps of the simulation procedure are listed below. Two different scenarios were considered to determine whether the fitted mixture model was affected by fiddling. If it is not affected by fiddling, then it serves as a useful model for computing expected numbers of p-values under the level 2 null case of no fiddling.

- Data for *N* tests are simulated as described earlier for a level 2 null case of no fiddling and a case in which fiddling has occurred (i.e., a level 2 alternative case). Denote the two resulting collections of p-values as P.null and P.alt, respectively.
- The mixture model is fit to the P.null p-values.
- Obtain the expected number of p-values as described in Step 3 above.
- Obtain the actual numbers of p-values, 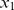 and 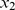, that are defined in Step 4 above, from both P.null and P.alt.
- Construct two contingency tables as described in Step 5 above and shown in Table 7, with one using the actual counts from P.null and one using the counts from P.alt. The counts from P.null will be used to assess type I errors, and the counts from P.alt will be used to evaluate power.
- Conduct the tests in Step 6 above.
- Repeat 1000 times and compute type I error and power at a level 2 rejection region of size 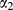.
- Repeat for number of studies (i.e., p-values) equal to *N = *400, 600, 800, 1000, and 2000.
- Redo the above Steps A–H, but modify Step B by fitting the mixture model to the collection of p-values from P.alt.

**Figure 2:**
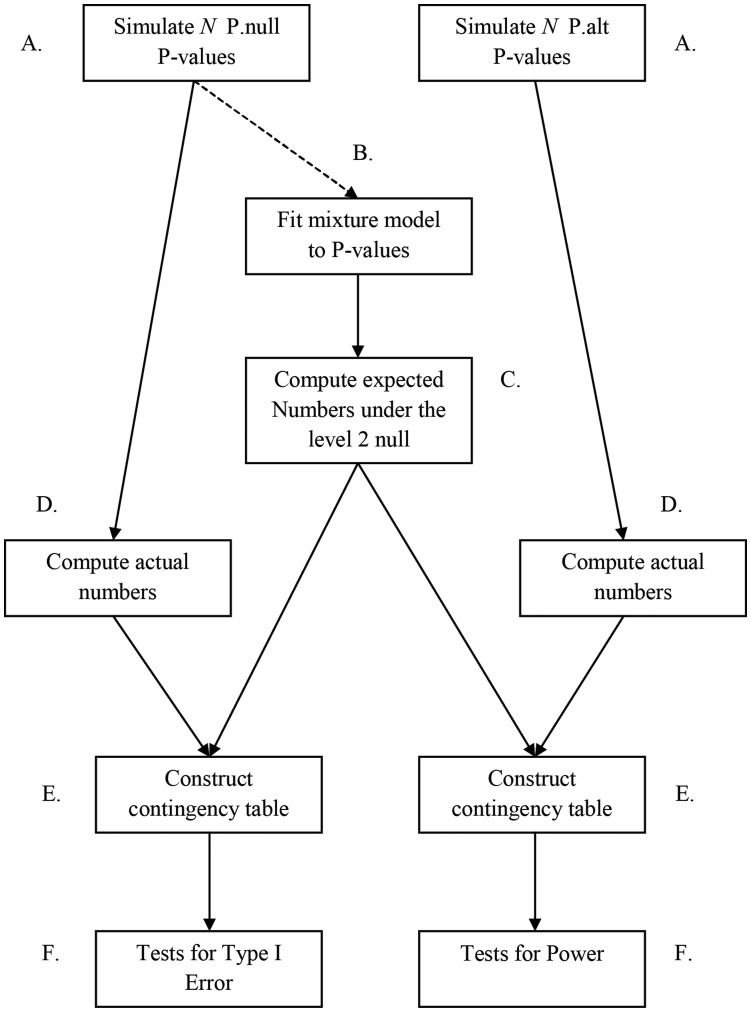
Steps A – F for the simulation procedure as described in section 5.3. The steps are repeated 1000 times for various sample sizes given in step H from section 5.3. For the final step I in section 5.3, the dashed line above for step B is redirected to instead connect the P.alt P-values to fitting the mixture model.

Figure 2 diagrams the flow of steps A – F with steps G, H, and I described in the caption to the figure. The results from these steps are reported in Table 8 for the scenario in which the mixture model was fit to the p-values from P.null from each simulated data set. The results are reported in Table 9 for the scenario in which the mixture model was fit to the p-values from P.alt obtained from each simulated data set. An adjustment was made to the size of the level 2 rejection region (denoted by 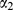
in Step G above) for the results reported in Tables 8 and 9. For a rejection region of size 0.05, the type I error rate for the tests based on the mixture model was much smaller than for the proportions tests described in section 4. This made a comparison of power between the two types of tests challenging. So the size of the rejection region was increased to 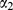 = 0.1 (in step G above) for the tests based on the mixture model. This resulted in an actual type I error rate for the mixture model approach that is similar to that for the tests of proportions in the prior section 4. So type I error rates are similar across Tables 6, 8, and 9, making power comparisons easier across these tables.

**Table 8:** Results for the Scenario in Which the Mixture Model was Fit to the p-Values From P.null^a^.

*N*	Type I Error (Chi-Sq Test)	Type I Error (Fisher’s Test)	Power (Chi-Sq Test)	Power (Fisher’s Test)
400	0.021	0.032	0.339	0.417
600	0.017	0.034	0.564	0.608
800	0.019	0.027	0.711	0.756
1000	0.022	0.029	0.791	0.817
2000	0.021	0.028	0.976	0.982

a Type I error and power are reported for two different tests for contingency table data.

**Table 9:** Results for the Scenario in Which the Mixture Model was Fit to the p-Values From P.alt^a^.

*N*	Type I Error (Chi-Sq Test)	Type I Error (Fisher’s Test)	Power (Chi-Sq Test)	Power (Fisher’s Test)
400	0.017	0.024	0.343	0.416
600	0.019	0.030	0.578	0.635
800	0.021	0.031	0.716	0.758
1000	0.024	0.031	0.817	0.842
2000	0.021	0.028	0.980	0.982

a Type I error and power are reported for various sample sizes for two different tests using contingency table data.

Several remarks can be made regarding the simulation results. First is that the type I error rate was below the nominal level of 0.05. This suggests that the fitted mixture model accurately predicted the number of p-values in the two intervals (0.05, 0.075] and (0.075, 0.1]. Moreover, for the purposes of type I error, it makes no difference whether the model is fit to p-values simulated with fiddling (P.alt) or with no fiddling (P.null). Similarly, if the mixture model is fit to either P.null or P.alt, the power of the test to detect fiddling when it has actually occurred is nearly the same. Power starts to rise to values generally considered acceptable for level 2 testing when the number of p-values exceeds 800. This suggests that, for a user who will not know whether fiddling has occurred in a practical application, the fitted mixture model is robust to this lack of knowledge. Power is somewhat higher for Fisher’s exact test versus the chi-square test, but the type I error rate is also slightly larger.

In conclusion, we have shown that tests of fiddling can be constructed and successfully applied to collections of p-values, which one can obtain from published literature. Different tests have been illustrated that all have acceptable level 2 type I error rates and have good power under realistic scenarios when the number of p-values available for analysis approaches 1000. These tests can be used going forward to study the extent to which fiddling seems to be occurring in the research literature and the factors associated with greater or lesser fiddling.

## Discussion

To summarize, we have shown that our proposed tests for fiddling can be successfully applied to vectors of p-values, which can be extracted from published papers. Several different tests were evaluated, and all had type I error rates at or below the nominal level and had good power in realistic circumstances, provided the number of p-values in the vector analyzed was near 1000. Our tests will be useful in the next stage of our research, wherein we will investigate the extent to which fiddling seems to be occurring in various research literature within the domains of nutrition and obesity and which factors are associated with the extent of fiddling. There are several points of discussion related to this particular endeavor or one similar to it.

On the surface, the topic of publication bias mentioned in the introduction may seem to be tightly related to the topic of fiddling discussed here. We generally think of publication bias as the tendency for statistically significant results to appear more frequently in literature versus non-significant results. The effect of this publication bias on the distribution of a collected set of p-values would be to steepen the descent of a curve that is fitted to a distribution of p-values as p-values go from 0 towards larger values – that is, publication bias in which significant findings are more likely to be published will only steepen the monotonic decreasing nature of the expected sampling distribution of observed p-values. The proposed mixture model will accommodate this. The mixture of a uniform and a two parameter beta distribution is quite flexible in capturing varying shapes on the interval 0 to 1. Fiddling, as we describe it, will produce a more distinct aberration in the distribution of p-values near 0.05 (or an alternative alpha level chosen). So even in the presence of publication bias, the mixture model should work in the same way that it is reported here. If the particular aberration is detected by our method, intuition would suggest that the detection is specific to fiddling. However, one could not be certain that the detection was not the result of some peculiar form of publication bias.

The relationship of fiddling to a ‘delayed analysis’ (i.e., waiting for a few more events to come in) is also worth some discussion. Let us consider that an investigator had a fixed number of cases preplanned for his/her study, repeatedly conducted significance tests as data accumulated, terminated the study either when the result was statistically significant or when the final sample size had been reached (whichever came first), and made no corrections for such repeated testing. Such procedures would increase type 1 error rates under the null hypothesis and increase power under the alternative hypothesis, but would not meet our definition of fiddling. Our definition of fiddling entails deciding to conduct additional testing only when p-values are just above the threshold for significance. Moreover, the procedure of repeated interim testing described above would not produce the characteristic dip in the distribution of p-values we have described.

In contrast, if an investigator collects an initial set of observations and then decides to collect an additional number of cases if and only if the initial result is just above the threshold of significance, then this is a form of fiddling as we have defined it and it would produce a ‘depletion’ of published p-values just-above the significance threshold. As discussed above in the comparison between publication bias versus fiddling, this ‘fiddled with result’ may then lead to a greater chance of publication as a result of publication bias.

If an effect is present but small, then collecting additional cases may lead to a statistically significant result (i.e., p-value <0.05) that is not of practical significance. Interval estimates of effect sizes can be as valuable (in some cases more valuable) as the p-values to which they correspond. As such, one might wonder how fiddling would be detected when effect sizes are reported in the literature in lieu of, or in addition to, p-values. The effect of fiddling on estimates of effect size should also produce a pattern if a sufficient number of interval estimates of effect sizes could be gathered. If, say, estimates are of contrasts and that contrasts not equal to zero are ‘significant,’ then fiddling would seem to produce an unusually large number of contrasts that narrowly miss covering zero. How to detect this aberration in interval estimates is not clear to us right now. Still, if fiddling is detected in a collection of p-values, a useful follow-up investigation would be to consider estimated effect sizes (if available) that correspond to p-values just below 0.05 and what proportion of those correspond to effects that are considered of practical significance for the particular application.

Another point of discussion is the seemingly large numbers of p-values that are needed for our proposed method to work effectively. Our method to detect fiddling requires a sufficient number of p-values in a narrow interval near some threshold (we used 0.05 as the most common threshold for ‘statistical significance’). Given that p-values can fall in the interval 0 to 1, the number of studies must be sufficiently large to produce a sufficient number of p-values in the subinterval 0.05 to 0.1 so that a test for fiddling can be carried out with reasonable power. Randomly selected p-values from, say, 20 studies may only produce as few as 1 or 2 p-values in the interval 0.05 to 0.1, and the test for fiddling as we propose it would have extremely little power. However, one could still proceed to conduct the binomial test for fiddling that is described in section 4 (test 2). If one could pre-screen literature to randomly select p-values near 0.05, then the binomial test as described in section 4 (test 2) could be carried out with a smaller sample of p-values while retaining an adequate level of power. So having adequate power to detect fiddling of p-values in a narrow subinterval near 0.05 is one reason why a sufficiently large number of p-values are needed. When using the mixture model approach from section 5, there is a second reason for requiring a large number of p-values. This reason relates to convergence of the optimization algorithm that produces maximum likelihood estimates of parameters in the mixture model. In our experience, for a particular analysis 100–200 p-values in the interval of 0 to 1 are sufficient to obtain convergence of the algorithm. With a particular analysis, the user has the luxury of tweaking starting values for the algorithm so that the chance of convergence is enhanced. This is not possible in simulations. The minimum we used in the simulation study to assure convergence in the 1000 simulations was 400 p-values.

We acknowledge that collecting such a large number of p-values entails considerable work and are experiencing that directly as we begin the next stage of our work as described in the first paragraph of this section. As also noted earlier, Ridley et al [4] used 3000 reported p-values. We currently have applied projects using this technique underway and have multiple students and trainees extracting p-values from hundreds of papers. For the future, natural language processing might be adapted to automatically search a body of literature and extract specific information relevant to a study as described herein. For the time being, however, we simply acknowledge as a challenge the large number of studies required for the detection of fiddling in the literature. It is unlikely that an individual investigator that is interested in whether fiddling is occurring in research would have sufficient interest, or the time, to carry out such an undertaking. An investigation of fiddling would more likely involve a team of researchers interested in studying research integrity and what factors play a role in buttressing research integrity. Some factors of interest in such a study might be tenured versus untenured research faculty, industry funded or non-industry funded research, before versus after the development of the CONSORT Guidelines, and so forth. Our proposed method for detecting fiddling could have utility in supporting such studies, and the studies would need to be sufficiently broad to allow for a large number of collected p-values. If a planned investigation into fiddling was too narrow, e.g., the effects of a very specific treatment on a very specific disorder, there may simply not be enough p-values for collection.

Given the labor intensive effort involved to conduct an investigation of fiddling, a clear plan for the investigation up front becomes vitally important. Some key questions to be considered in doing so are: 1) How should one best extract p-values from the literature?; 2) Which areas of study and/or which publications and years should be considered? (For example, in some areas of genomics, alpha levels typically are set at levels between 10^−4^ and 10^−8^, rather than 0.05); and 3) How should rounded p-values be used if used at all? Ridley et al. [4] dealt with these questions to some extent. For instance, they used raw reported p-values from three identified prominent journals covering two years and ignored rounded p-values.

In addition to the above questions dealing with technical logistics for a general investigation of fiddling, more specific scientific questions that are of interest can also shape the plan and conduct of the investigation. Such questions are, 1) Are ‘fiddling’ rates higher in high profile journals that are harder to get published in versus lower profile journals? 2) Has the desire to ‘fiddle’ changed over the years and what time-frame should suitable papers be selected from? 3) Does ‘fiddling’ vary by subject area? In answering question 1, an analysis stratified by publication may need to be considered. Or separate analyses of fiddling for each journal conducted separately. Since the proposed tests for fiddling use data in two by two contingency tables, each test corresponds to a test of a difference in two proportions. As such, an interval estimate of this difference can be obtained and ‘significant differences’ in effect sizes of fiddling in one journal (or time period) versus another journal (or time period) can be compared. These and other interesting applied questions can be addressed going forward using the method we have offered herein. By doing so, we hope that areas in which research practices can be improved can be identified and constructive feedback provided to the field.
